# Decreasing Mob Size at Lambing Increases the Survival of Triplet Lambs Born on Farms across Southern Australia

**DOI:** 10.3390/ani13121936

**Published:** 2023-06-09

**Authors:** Amy Lockwood, Travis Allington, Sarah E. Blumer, Johan Boshoff, Bronwyn E. Clarke, Serina N. Hancock, Gavin A. Kearney, Paul R. Kenyon, Jarryd Krog, Lyndon J. Kubeil, Gordon Refshauge, Jason P. Trompf, Andrew N. Thompson

**Affiliations:** 1 Centre for Animal Production and Health, Murdoch University, Perth, WA 6150, Australia; 2 Faculty of Science, Agriculture, Business and Law, University of New England, Elm Avenue, Armidale, NSW 2351, Australia; 3 36 Paynes Road, Hamilton, VIC 3300, Australia; 4 School of Agriculture and Environment, Massey University, Tennent Drive, Palmerston North 4410, New Zealand; 5 Department of Economic Development, Jobs, Transport and Resources, 89 Sydney Road, Benalla, VIC 3672, Australia; 6 New South Wales Department of Primary Industries, Cowra Agricultural Research and Advisory Station, 296 Binni Creek Road, Cowra, NSW 2794, Australia; 7 J.T. Agri-Source, 2A Bradley Drive, Melbourne, VIC 3082, Australia

**Keywords:** mob size, lamb survival, triplet, triplet-bearing ewes, triplet-born lambs, Merino, maternal, non-Merino

## Abstract

**Simple Summary:**

Consultation with sheep producers in Australia revealed that understanding the impact of the number of triplet-bearing ewes in a paddock at lambing, known as the mob size, on the survival of their lambs was an important research priority. Previous research has demonstrated that smaller mob sizes at lambing improve the survival of single- and especially twin-born lambs. Therefore, we expected that lambing triplet-bearing ewes in smaller mobs would increase the survival of their lambs. Research was conducted on 12 commercial sheep farms across southern Australia between 2019 and 2021, with three farms used in two years of the experiment. Adult, triplet-bearing ewes were randomly allocated into one of two treatments, ‘High’ or ‘Low’ mob size, at about 15 days before the start of lambing. We found that lamb survival was significantly greater for lambs born in the Low compared with the High mob size treatments. Analysis of the effect of the actual mob sizes showed that reducing mob size at lambing by 10 triplet-bearing ewes increased the survival of their lambs by 1.5%. Lambing triplet-bearing ewes in smaller mobs will therefore be included in management guidelines for producers as a strategy to improve the survival of triplet-born lambs.

**Abstract:**

Industry consultation in Australia revealed that the potential impact of the mob size of ewes during lambing on the survival of triplet-born lambs was an important research priority. Previous research has demonstrated that smaller mob sizes at lambing improve the survival of single- and especially twin-born lambs, regardless of ewe stocking rate. Therefore, we hypothesised that lambing triplet-bearing ewes in smaller mobs, regardless of stocking rate, will increase the survival of their lambs. Research sites were established on 12 commercial sheep farms across southern Australia between 2019 and 2021. One farm used Merinos whilst the remainder of the farms used non-Merino breeds, consisting of composite ewes joined to composite or terminal sires. Three of the farms were used in two years of the experiment. Adult, triplet-bearing ewes were randomly allocated into one of two treatments, ‘High’ or ‘Low’ mob size, at an average of 135 days from the start of joining. Ewe and lamb survival were assessed between allocation to treatments and lamb marking. Lamb survival was significantly greater for lambs born in the Low (65.6%) compared with the High (56.6%) mob size treatments (*p* < 0.001). There was no effect of mob size at lambing on the mortality of triplet-bearing ewes. Analysis of the effect of the actual mob sizes showed that reducing the mob size at lambing by 10 triplet-bearing ewes increased the survival of their lambs to marking by 1.5% (*p* < 0.001). This study has shown that the survival of triplet-born lambs can be improved by lambing triplet-bearing ewes in smaller mobs regardless of stocking rate when ranging from 0.7–13 ewes/ha.

## 1. Introduction

The profitability of sheep enterprises can be improved by increasing the survival of lambs to marking, especially for non-Merino sheep and when meat prices are high [1]. Marking rates, or the number of lambs marked per 100 ewes joined, increased from about 81% to 92%, or by about 1% per annum, between 2006 and 2016 in Australia [2]. Increased marking rates have resulted from a combination of strategies, including better ewe nutrition before joining and during pregnancy, improved management during lambing, and genetic selection for fertility, fecundity or traits related to lamb survival [3,4,5]. In addition, there has been a significant displacement of Merino sheep with more fecund maternal ewe types, which consistently mark at least 20% more lambs [2]. Increased fecundity is associated with a greater proportion of multiple-bearing ewes, including those carrying triplets, which can result in higher rates of mortality in both ewes and lambs [6,7]. The high rates of mortality in triplet-bearing ewes and their lambs limit potential gains in productivity and represent an animal welfare concern.

Industry consultation in Australia revealed that the potential impact of the mob size of ewes during lambing on the survival of triplet-born lambs was an important research priority [7]. Past survey data collected from sheep producers in south-eastern Australia found that the survival of single- and twin-born lambs increased by 1.4% and 3.5% when the mob size at lambing was reduced by 100 ewes [8]. This was verified by experimental data collected from 70 on-farm research sites across Australia, which found that reducing the mob size at lambing by 100 twin-bearing ewes increased the survival of their lambs by 1.9%, regardless of the breed and stocking rate at lambing [9]. Similarly, data collected from 15 on-farm research sites found that reducing the mob size at lambing by 100 twin-bearing Merino ewes increased the survival of their lambs by 2.5% when ewes lambed at stocking rates of less than 4 ewes/ha [9]. Economic analysis has been conducted to assess the optimum mob size at lambing for single- and twin-bearing ewes. This demonstrated that no generic recommendations can be made, as the optimum mob size is dependent on several enterprise-specific factors. However, the optimum mob size for twin-bearing ewes was smaller than single-bearing ewes by approximately 55% for Merinos and 62% for non-Merino ewes [10]. Therefore, it was concluded that smaller mobs at lambing should be prioritised for twin-bearing ewes over single-bearing ewes.

There has been no experimental work conducted to understand the impacts of mob size at lambing on triplet-bearing ewes and their lambs. The relationship between mob size and lamb survival is understood to be largely driven by the risk of mismothering [11,12]. Triplet-born lambs are poorer at identifying their dam than twin-born lambs [13]. Triplet-bearing ewes also display limited bleating when separated from one of their lambs, and are less likely to approach and reunite with the lamb [13]. Similarly, Cloete [11] found that the risk of ewe-lamb separation was greater for triplets and twins compared with singles, and increased with a greater density of lambed ewes. It is possible that disturbances during labour may also prolong delivery and increase the risk of dystocia, ewe exhaustion and mismothering. Triplet-bearing Merino ewes have been observed to take longer to deliver their litter than twin- and single-bearing ewes [11], and triplet-born lambs are at greater risk of death due to dystocia than single- and twin-born lambs [14]. In addition, more triplet lambs will be born per day in the same mob size when compared to twins or singles. Therefore, there is significant opportunity for triplet-bearing ewes and their lambs to be disturbed during the periparturient period. The poorer behaviour of triplet-born lambs, including taking longer to stand [15] and suckle after birth [15,16,17], would be expected to further increase the risk of mismothering. Therefore, we hypothesise that lambing triplet-bearing ewes in smaller mobs, regardless of stocking rate, will increase the survival of their lambs.

## 2. Materials and Methods

### 2.1. Research Site, Animals and Experimental Design

**Figure 1:**
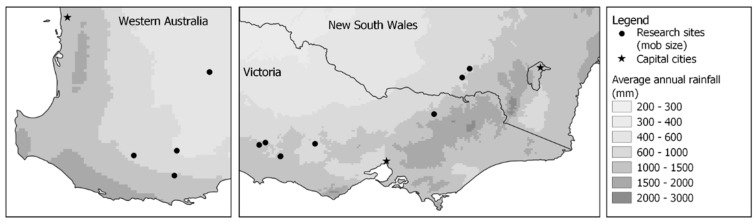
Location and average annual rainfall of on-farm research sites across Western Australia, Victoria and New South Wales. Data for annual rainfall were obtained from the Australian Government Bureau of Meteorology.

Research sites were established on 12 commercial sheep farms across Western Australia (*n* = 4), Victoria (*n* = 7) and New South Wales (*n* = 1) between 2019 and 2021. One farm used Merinos whilst the remainder of the farms used non-Merino breeds, consisting of composite ewes joined to composite or terminal sires. Three of the farms were used in two years of the experiment; one farm with Merinos in Western Australia, one farm with non-Merinos in Western Australia and one farm with non-Merinos in Victoria. The locations of the research sites are shown in Figure 1. Adult, triplet-bearing ewes at each research site were randomly allocated into one of two treatments, High or Low mob size, at an average of 135 days from the start of joining. The aim was for the mob sizes of the High and Low treatments to differ by at least 40 ewes within each research site. Treatments at each research site were replicated where adequate ewes and paddocks were available. Four research sites did not include replication, whilst the remaining sites had one to four replicates of the High mob size treatment and two to six replicates of the Low mob size treatment. The average mob size of triplet-bearing ewes at lambing was 63 ewes for the High treatment and 20 ewes for the Low treatment, with an average difference of 40 ewes between the treatments at each research site. Paddock selection within the research sites aimed for ewes in the High and Low mob size treatments to be lambed at a similar stocking rate of within 2 ewes/ha. The mean and range for the stocking rate during lambing across all sites are shown in Table 1. Lambs were born between late autumn and spring.

### 2.2. Animal and Paddock Measurements

Ewes were joined for an average of 32 days across the research sites (range 15–37 days). Triplet-bearing ewes were managed as one group following pregnancy scanning until they were allocated to treatments. All ewes were condition scored at an average of 135 days from the start of joining before being randomly allocated to their treatments. Ewes were then moved to their allocated lambing paddocks, where they remained until lamb marking. Ewes were individually condition scored and assessed for lactation status (lactating or not) at lamb marking, at an average of 159 days from the end of joining. Ewes were condition scored by a single assessor at each research site when allocated to treatments and at lamb marking using 0.25 score increments on a scale of 1 to 5, as described by Jefferies [18]. The mean condition scores of the ewes at lambing and marking are shown in Table 1.

Management at each research site aimed for the quantity (feed-on-offer; FOO) and quality of pasture within the paddock to be similar for both treatments. Lambing paddocks on each farm were also selected to have similar characteristics. Paddock characteristics were recorded by a single assessor at each research site and included paddock shape, topography, the number and type of watering points and shelter availability. The characterisation of paddock topography and shelter availability were as described by Lockwood et al. [9]. Most (70%) of the paddocks were square or rectangular with flat to gently undulating topography. Shelter in 96% of the paddocks was provided by trees or tall shrubs of varying density and distribution. The mean availability of shelter is shown in Table 1.

### 2.3. Assessment of Feed-on-Offer and Pasture Composition

Visual estimates of FOO (kg DM/ha) were assessed at 25 sites in each paddock on day 140 from the start of joining and at lamb marking by the same assessor at each research site. The percentage of legume in the pasture was also assessed at the same sites. Visual estimates of FOO were calibrated against 10 quadrat cuts, as described by Lockwood et al. [9]. The mean and ranges for FOO at lambing and the proportion of legume in the pasture across all research sites are shown in Table 1.

### 2.4. Weather Conditions during Lambing

Daily data for temperature, rainfall and windspeed between day 145 from the start of lambing and lamb marking were collected via the Australian Gridded Climate Data (AGCD) and Australian Community Climate and Earth-System Simulator (ACCESS-G) services from the Australian Government Bureau of Meteorology for each research site. Windspeed at 10 m was provided by the Bureau of Meteorology and was converted to a lamb height of 0.4 m using the formula described by Thornley and Johnson [19]. Daily chill index was calculated for each research site using the formula described by Nixon-Smith [20], with the weighting of the daily temperature (0.75 × maximum temperature + 0.25 × minimum temperature) as per Horton et al. [21]. The mean chill index between day 145 from the start of joining and lamb marking was then calculated. The mean chill index during lambing across all research sites was 759 kJ/m^2^/h, with a range in means of 672 to 814 kJ/m^2^/h.

### 2.5. Statistical Analysis

Lamb survival for each mob was calculated based on the number of foetuses identified in ewes at pregnancy scanning and the number of lambs marked. Ewe mortality for each mob was calculated based on the number of ewes present when allocated to treatments and the number of ewes present at lamb marking.

Lamb survival and ewe mortality were analysed using the method of restricted maximum likelihood in GENSTAT (VSN International 2017). The mob size treatments (high or low) and covariates, including the average condition score of ewes at lambing, FOO at lambing, the proportion of legume in the pasture at lambing, shelter availability within the lambing paddock and the average chill index during lambing, were fitted as fixed effects, while year, farm (nested within year) and paddock (nested within farm) were fitted as random effects. Further analysis examined the actual effect of mob size given that the treatment effect was significant. This was performed using restricted maximum likelihood and fitting the actual ewe mob size as a fixed effect, while year, farm (nested within year) and paddock (nested within farm) were fitted as random effects. All possible models were examined with the statistical significance of terms and interactions thereof accepted at *p* < 0.05.

## 3. Results

**Figure 2:**
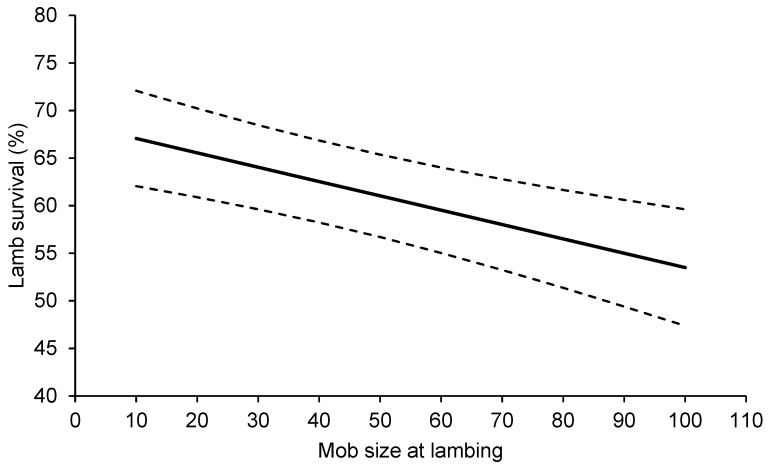
The effect (±95% confidence intervals; dashed lines) of the mob size of triplet-bearing ewes of Merino and non-Merino breeds at lambing on the survival of their lambs to marking at research sites across southern Australia between 2019 and 2021.

There was no effect of ewe condition score at lambing (*p* > 0.149), the stocking rate of ewes at lambing (*p* > 0.751), FOO at lambing (*p* > 0.245), the proportion of legume in the pasture at lambing (*p* > 0.258), shelter availability within the lambing paddock (*p* > 0.151) or the average chill index during lambing (*p* > 0.476) on lamb survival or ewe mortality, nor any interaction with treatment. Therefore, these terms were removed from the statistical model. Lamb survival was significantly greater for lambs born in the Low compared with the High mob size treatments (Table 2). There was no effect of mob size at lambing on the mortality of triplet-bearing ewes (Table 2). Analysis of the effect of the actual mob sizes showed that reducing the mob size at lambing by 10 triplet-bearing ewes increased the survival of their lambs to marking by 1.5% (Figure 2; *p* < 0.001).

## 4. Discussion

Reducing the mob size at lambing by 10 triplet-bearing ewes increased the survival of their lambs by 1.5% when the mob size ranged from 10 to 139 triplet-bearing ewes and the stocking rate ranged from 0.7 to 13.4 ewes/ha. Therefore, our hypothesis was supported. The magnitude of the impact of mob size on the survival of triplet-born lambs was 4- to 8-fold greater than that observed for twin-born lambs in Australia by Lockwood et al. [8,9]. Albeit, the average mob size examined in the current study was much smaller and covered a lower range than that in the studies of Lockwood et al. [8,9]. The limited adoption of pregnancy scanning for triplets means that there are little data available regarding the mob size at which triplet-bearing ewes are lambed at commercial enterprises in Australia. However, it appears that the range in mob size and stocking rate assessed in our study reflects commercial management [7,22]. The ewes in our study were mostly of non-Merino breeds and therefore further experimentation using Merino ewes is warranted in order to investigate whether there is an impact of ewe breed on the relationship between the mob size of triplet-bearing ewes at lambing and the survival of their lambs. Economic analysis is also required to examine the optimum mob sizes for triplet-bearing ewes and the value of paddock subdivision to lamb ewes in smaller mobs. Triplet-bearing ewes may only represent a small proportion of pregnant ewes within the enterprise, particularly for Merinos, and therefore balancing the allocation of resources, mob sizes and paddocks for twin- and triplet-bearing ewes is important to improving the overall marking rates for the enterprise.

Lamb survival and the impact of the mob size of triplet-bearing ewes at lambing on lamb survival were not influenced by ewe stocking rates of up to 13 ewes/ha at lambing, FOO at lambing ranging from 270 to 2140 kg DM/ha, the proportion of legume within the pasture at lambing or the characteristics of the lambing paddock. These findings are consistent with those observed for twin-bearing ewes and their lambs by Lockwood et al. [9], noting that the mob sizes for twin-bearing ewes were larger, on average, than those for triplet-bearing ewes. However, the findings of Lockwood et al. [23] suggested that the mob size of twin-bearing ewes at lambing may have no effect on lamb survival when FOO at lambing exceeds 2400 kg DM/ha. In contrast, Lockwood et al. [24] observed that the mob size of twin-bearing ewes at lambing had a significant effect on lamb survival when FOO was limited (<390 kg DM/ha) and ewes were supplementary fed during lambing. These findings contrast with those of the current study and of Lockwood et al. [9], and highlight that further work is required to investigate the relationship between FOO, supplementary feeding and the mob size at lambing on lamb survival. The ewe condition score at lambing was not found to influence lamb survival in the current study. This contrasts the findings of some studies that have demonstrated a negative relationship between the ewe condition score at lambing and the survival of lambs born to triplet-bearing Merino ewes [25] or multiple-bearing Maternal ewes, including twin- and triplet-bearing ewes [3,26]. However, the average condition score of ewes was just above three and therefore the ewe condition score may have been close to optimal for lamb survival. Most ewes were also non-Merino in our study, and recent work by Haslin et al. [25] found no difference in the survival of triplet lambs born to ewes, with an average condition score of 3.1 versus 3.5 at lambing.

Shelter had no signficant effect on lamb survival in this study. Weather conditions at lambing were relatively mild across the research sites, with the average chill index during lambing ranging from 672 to 814 kJ/m^2^/h. Lamb mortality is typically greatest when the chill index exceeds 1000 kJ/m^2^/h, however lambs with a lower birthweight can be susceptible at lower indices [21]. Triplet lambs are typically born at lower birthweights than single- and twin-born lambs and are therefore most vulnerable to hypothermia and thus most likely to benefit from the provision of shelter [6]. The economic pay-off of providing shelter to reduce the mortality of twin-born lambs is greater in environments that regularly experience high-chill conditions during lambing [27]. It would therefore be expected that the economic benefits of providing shelter for triplet-born lambs will also be greatest under high-chill conditions, which, on average, were not observed during lambing in this study. Further work is required to understand the impacts of shelter availability on the survival of triplet-born lambs, including the efficacy of different types and designs of shelter within the paddock, and the utilisation of the shelter by lambing ewes and newborn lambs.

The mortality of ewes in the current study was similar to that recently reported by Thompson et al. [7] and Haslin et al. [25]. It was not surprising that the mob size of triplet-bearing ewes at lambing had no significant effect on ewe survival. The impacts of the mob size at lambing are believed to be largely exerted on the lambs due to an increased risk of mismothering. Notably, mismothering is perceived by Australian producers to be the greatest cause of death of triplet-born lambs [7]. It is possible that greater mob sizes could increase interference between periparturient ewes due to their attraction to amniotic fluids and newborn lambs. Increased interference during labour has the potential to increase the risk of dystocia [28] and this may subsequently increase the risk of ewe mortality. Previous research with single- and twin-bearing ewes has shown no effect of High or Low mob size treatments on the incidence of lamb mortality due to dystocia, but the effect on ewe mortality was not investigated [23,24]. While the incidence of dystocia in ewes was also not recorded in the current study, the average condition score of 3.1–3.2 for ewes at lambing likely reduced the risk of dystocia due to low- or high-birthweight lambs or that associated with ewe health issues due to low or excessive condition scores [28]. The relatively small mob sizes within this study may have also limited the likelihood of interference between periparturient ewes due to a smaller number of ewes lambing at a similar time when compared with larger mobs. Overall, our results imply that the mob size of triplet-bearing ewes is important for the survival of their lambs but not for the survival of the ewes. However, further work is warranted to investigate the cause of death of triplet-bearing ewes and their lambs to enable comprehensive management guidelines to be developed to improve survival and welfare.

## 5. Conclusions

This study has shown that the survival of triplet-born lambs can be improved by lambing triplet-bearing ewes in smaller mobs regardless of stocking rate, when ranging up to 13 ewes/ha, under extensive conditions in southern Australia. Economic analysis is required to determine the optimum mob sizes at lambing for triplet-bearing ewes and whether the permanent or temporary subdivision of paddocks is profitable for producers to achieve these smaller mob sizes. Overall, this work will contribute to the development of best-practice management guidelines for producers to improve the reproductive performance and welfare of their triplet-bearing ewes and lambs.

## Figures and Tables

**Table 1:** Number of mobs and the mean (range) for stocking rate (ewes/ha) at lambing, ewe condition score at lambing, feed-on-offer (kg DM/ha) at lambing, proportion of legume in the pasture at lambing (%) and percentage of the lambing paddock with shelter for the High and Low mob size treatments at research sites across Australia between 2019 and 2021.

Treatment	*n* Mobs	Stocking Rate	Condition Score at Lambing	Feed-On-Offer ^1^ at Lambing	Legume	Shelter ^2^
High mob size	31	5.1	3.1	1110	34	10
		(0.7–13.4)	(2.5–3.7)	(270–1790)	(0–90)	(0–30)
Low mob size	47	5.0	3.2	1210	26	7
		(0.7–11.2)	(2.6–3.7)	(370–2140)	(0–76)	(0–20)

^1^ Feed-on-offer refers to the quantity of pasture within the paddock. ^2^ The shelter percentage relates to shelter within the paddock boundary.

**Table 2:** Mortality (%) of ewes of Merino and non-Merino breeds between allocation to treatments at an average of 135 days from the start of joining and lamb marking, and survival (%) of their lambs to marking for the High and Low mob size treatments at research sites across Australia between 2019 and 2021.

	High Mob Size	Low Mob Size	l.s.d.	*p*-Value
Ewe mortality	6.3	5.1	1.68	0.179
Lamb survival	56.6	65.6	3.62	<0.001

## Data Availability

The datasets generated and/or analysed during the current study are not publicly available but are available from the corresponding author on reasonable request pending permission from the funding body (Meat and Livestock Australia) and Murdoch University.
